# Alleviating symptoms of paediatric acute rhinosinusitis and acute otitis media with otorrhea using nasal-spraying *Bacillus* probiotics: a randomized controlled trial

**DOI:** 10.1038/s41598-025-87372-2

**Published:** 2025-01-27

**Authors:** Thanh Huu Khieu, Dung Phuong Le, Binh Thanh Nguyen, Binh Thanh Ngo, Hong Thi Chu, Duc Minh Truong, Hung Minh Nguyen, Anh Hoa Nguyen, Tung Dinh Pham, Anh Thi Van Nguyen

**Affiliations:** 1 Thai Binh University of Medicine and Pharmacy, 373 Ly Bon, Thai Binh, Vietnam; 2 Department of Otolaryngology, Thai Binh Medical University Hospital, 373 Ly Bon, Thai Binh, Vietnam; 3 Thai Binh Pediatric Hospital, 2 Ton That Tung, Thai Binh, Vietnam; 4 Spobiotic Research Center, ANABIO R&D Ltd. Company, No. 22, Lot 7,8 Van Khe Urban, La Khe, Ha Dong, Hanoi, Vietnam; 5 LiveSpo Pharma Ltd. Company, N03T5, Ngoai Giao Doan Urban, Bac Tu Liem, Hanoi, Vietnam; 6 Faculty of Mathematics - Mechanics - Informatics, VNU University of Science, Vietnam National University_Hanoi, 334 Nguyen Trai, Thanh Xuan, Hanoi, Vietnam

**Keywords:** Acute, Rhinosinusitis, Otitis media, Nasal-spraying *Bacillus* spores, Bacterial co-infection, Cytokine, Randomized controlled trials, Respiratory signs and symptoms, Paediatric research, Interleukins, Clinical microbiology

## Abstract

Acute rhinosinusitis (ARS) in children may be accompanied by acute otitis media (AOM) which is often associated with bacterial co-infections. These conditions are among the primary reasons that children visit hospitals and require antibiotic treatment. This study evaluated the efficacy of the nasal-spraying probiotics (LiveSpo Navax containing 5 billion *Bacillus subtilis* and *B. clausii* spores/5 mL) as a supportive treatment for dual ARS and AOM with otorrhea in a randomized, single-blind, controlled clinical trial. Eighty-two patients (41 per group), aged 1 month to 12 years, received standard care along with nasal spraying of either physiological saline (Control group) or LiveSpo Navax (Navax group), administered three times daily over a 7-day follow-up period. A total of sixty-one patients (30–31 per group) completed the trial. The Navax group experienced 68.00% and 96.77% reductions in nasal congestion (by day 3) and rhinorrhea (by day 7), respectively, which were 2.04 and 1.94-fold higher than the Control group, with odds ratios (OR) of 4.31 and 30.00 (*p* < 0.05). Endoscopic results indicated 8% and 11% higher reductions in nasal mucopurulent discharge and tympanic membrane hyperemia in the Navax group compared to the Control group. By day 3, compared to day 0, the Navax group exhibited > 1200-fold reduction in *Streptococcus pneumoniae* and ≥ 4-fold reduction in *Haemophilus influenzae* concentrations (*p* < 0.05) in both nasopharyngeal and middle ear fluid samples, whereas the Control group showed no significant reductions. Navax treatment reduced IL-6 by 1.35- to 1.74-fold and TNF-α by 1.17- to 1.45-fold, more effectively than the Control group (*p* < 0.05). These results suggest that nasal-spray *Bacillus* spore probiotics, with their ability to reduce bacterial load and modulate immune responses, provide a cost-effective and safe solution for alleviating symptoms of both ARS and AOM in children.

*Trial registration*: ClinicalTrials.gov, Identifier NCT05804123 on April 7, 2023.

**Supplementary Information:**

The online version contains supplementary material available at 10.1038/s41598-025-87372-2.

## Introduction

Acute otitis media (AOM), the most common ear condition in children, affects 90% of those under age two and 80% of preschoolers, with an incidence declining to 8% at age eight^1–3^. AOM complications including chronic suppurative otitis media and perforated tympanic membrane (TM) may occur in about 4.8% of cases at ages under five^1,4^. Typical symptoms of acute middle ear inflammation in AOM include fever, ear pain (otalgia), ear discharge (otorrhea), loss of appetite, temporary hearing loss, and sometime vomiting or diarrhea^1^. AOM can be caused by viruses such as respiratory syncytial virus (RSV), rhinovirus, influenza virus, and adenovirus, as well as co-infecting bacteria species associated with acute rhinosinusitis (ARS)^5–7^.

ARS is characterized by inflammation of the nasal cavity and sinus linings due to infection^8,9^. ARS in children is typically diagnosed with clinical symptoms and its duration of less than 12 weeks, often following an upper respiratory viral infection, with common symptoms include nasal blockage/obstruction/congestion, runny nose (rhinorrhea), discolored nasal discharge, cough, and even facial pain^10^. Acute bacterial rhinosinusitis is identified in 6–7% of children who exhibit upper respiratory tract symptoms in primary care clinics^9,10^. Between 5% and 10% of viral upper respiratory tract infections in children progress to acute bacterial rhinosinusitis. An association exists between ARS and AOM, involving concordance in the microbiologic findings^11^. The anatomical proximity of the nasopharynx and the ears is believed to allow infection to spread, facilitated by dysfunction of mucociliary clearance and the Eustachian tube^12^. The main bacterial pathogens responsible for both ARS and AOM include *Streptococcus pneumoniae*, *Haemophilus influenzae*, and *Moraxella catarrhalis*^6–9,11,12^.

Globally, most individuals with ARS and AOM are prescribed antibiotics^13–17^. Nonetheless, the efficacy of antibiotics in addressing both these diseases remains restricted. While antibiotics provide modest symptom relief for rhinosinusitis, most cases would resolve within two weeks, even without antibiotic intervention^18,19^. Importantly, antibiotic overuse in these common pediatric conditions may contribute to the risk of emerging antibiotic resistance at both the community and individual levels, prompting investigations for alternative approaches^20,21^. In particular, as major pathogens in both ARS and AOM, *S. pneumoniae* and *H. influenzae* are listed on the priority group 3 by WHO, due to their penicillin-non-susceptibility and ampicillin-resistance^22^.

Probiotics provide a promising approach for reducing respiratory bacterial infections while addressing the global challenge of antibiotic resistance. By avoiding adverse effects associated with antibiotics, such as microbiota disruption and secondary infections, probiotics offer a safer, natural alternative. Their mechanisms involve restoring microbiota balance and enhance the body’s defense mechanisms, which is particularly important for children with developing immune systems^23,24^. Thus, strains belonging to species such as *Lactobacillus rhamnosus*, *Enterococcus faecalis*, and *Lactococcus lactis* were reported to alleviate ARS symptoms and reduce relapses, highlighting their potential as alternative therapies^25–27^. Furthermore, a review of meta-analyses based on 17 randomized controlled trials has suggested that probiotics, particularly those containing *Lactobacillus* strains, may reduce the incidence of AOM and lower the use of antibiotics for infections. Nevertheless, the inconsistency in subgroup analyses and the variability in probiotic strains, dosages, frequencies, and durations across trials limit the generalizability of the findings^28^. Importantly, the effects of tested probiotics usually emerge gradually, often requiring at least two weeks of use or even months^25–27,29–32^, a delay that may originate from the oral administration route or the instability of non-spore-forming bacterial strains in liquid suspensions when used as nasal drops.

*Bacillus* species such as *B. subtilis* and *B. clausii* can maintain their effectiveness over extended periods due to their ability to produce heat-resistant spores even in nutrient-free aqueous solutions^33^. Our hypothesis is that when sprayed into the nose, a stable liquid-form *Bacillus* spore probiotics enable the spores to adhere to the nasal mucosa, and then migrate to the sinuses and middle ear via the Eustachian tube. Recent studies on nasal-spraying *Bacillus* spore probiotics (LiveSpo Navax) have shown their promising results in reducing the viral load, bacterial concentration, and inflammation in respiratory infections due to respiratory syncytial virus (RSV)^34^ and influenza virus^35^. Given the above hypothesis and recent findings, this liquid-form *Bacillus* spore probiotics may also be effective in alleviating symptoms in patients with ARS and AOM caused by bacterial infections. Therefore, in this single-blind, randomized, controlled clinical study, we evaluated the efficacy of nasal-spraying LiveSpo Navax for the symptomatic treatment of ARS and AOM in pediatric patients. Additionally, we assessed changes in co-infecting bacterial concentrations and immune indicators in nasopharyngeal and middle ear fluid samples before treatment (day 0) and during treatment (days 3 and 7) to investigate the mechanism of action behind these effects.

## Results

### Trial design and patient baseline demographic, clinical and sub-clinical characteristics

**Fig. 1:**
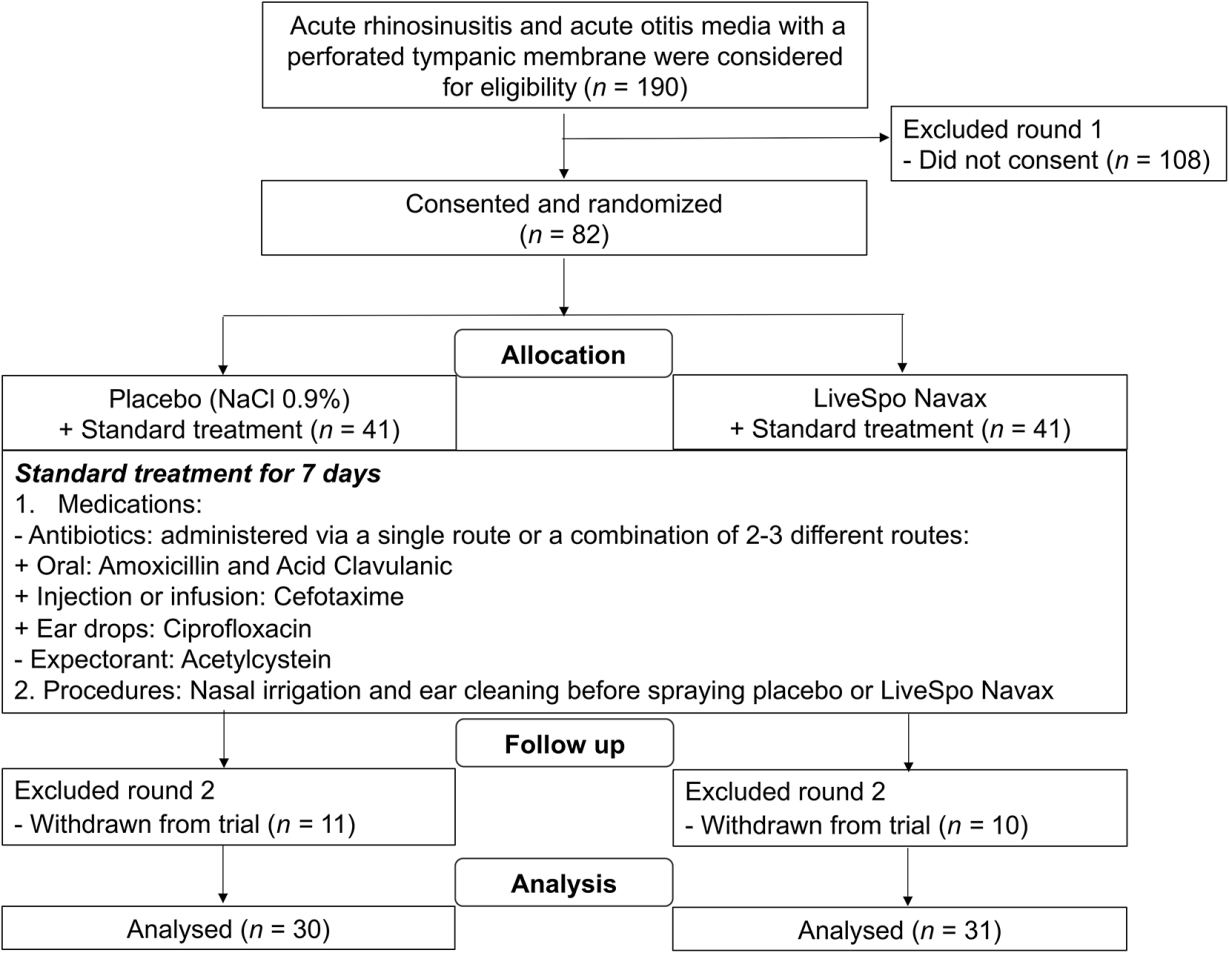
A CONSORT flowchart illustrating the patient recruitment and allocation process adopted in this study. It outlines the recruitment and allocation of participants, the standard treatment of care, follow-up, and analysis at the endpoint, conducted between July 2022 and March 2024.

A total of 190 patients who met the eligibility criteria for ARS and AOM with perforated TM were screened from August 2021 to March 2024. Among them, parents of 82 patients consented to participate in the study (Fig. 1, Excluded Round 1) and were then randomly assigned (1:1, *n* = 41 per group) to two groups: Control (received NaCl 0.9%) and Navax (received LiveSpo Navax). During the treatment period, which lasted 7 days, 11 patients from the Control group and 10 from the Navax group were withdrawn from the trial (Fig. 1, Excluded Round 2). Consequently, the final analysis at the end point included 30 patients in the Control group and 31 in the Navax group.

As shown in Table 1, the evaluation of age, gender, and clinical symptoms of rhinosinusitis and otitis media prior to study participation revealed no significant differences between the two groups (*p* > 0.05). In children with concurrent rhinosinusitis and otitis media, the predominant symptoms of rhinosinusitis included rhinorrhea (100%) and nasal congestion (> 80%), with few or no other symptoms observed (*p* > 0.05). The rate of typical symptoms of otitis media, such as otorrhea and earache, were high in both study groups, accounting for 87-100% of the participants. Other symptoms, including fever, poor response to sound, vomiting or diarrhea, and loss of appetite, were observed at lower rates, ranging from 30 to 55%. Endoscopic results revealed that all patients exhibited typical signs of rhinosinusitis, including mucosal edema and mucopurulent discharge from middle meatus. For otitis media, the most characteristic sign was purulent discharge through a perforated TM (100%), followed by TM hyperemia (> 96%). Other signs, such as swelling, cloudiness, and loss of the bright triangle in the TM, were observed in approximately 45–57% of the cases. The bacterial test results indicated that the three bacterial species *S. pneumoniae*, *H. influenzae*, and *M. catarrhalis* were detected at similar rates in both groups, with no statistically significant differences (*p* > 0.05). The rate of co-infection with 2 or 3 types of bacteria was also comparable between the groups (*p* > 0.05). In both groups, the rates of *S. pneumoniae* and *H. influenzae* infections were relatively high (32–45%), whereas the rates of *M. catarrhalis* infections were much lower (< 7%).

**Table 1:** Demographic, clinical and sub-clinical characteristics of patients before treatment.

Characteristics	Acute rhinosinusitis and otitis media		*p* value
	Control group (*N* = 30)	Navax group (*N* = 31)	
Age			
1 month to < 24 months *n* (%)	15 (50.00)	20 (64.52)	0.25^a^
≥ 2 years to 12 years old *n* (%)	15 (50.00)	11 (35.48)	
Median age (months)	23	20	
Gender			
Male *n* (%)	18 (60.00)	17 (54.84)	0.68^a^
Female *n* (%)	12 (40.00)	14 (45.16)	
Clinical symptoms of rhinosinusitis			
Nasal congestion *n* (%)	27 (90.00)	25 (80.65)	0.47^b^
Runny nose (rhinorrhea) *n* (%)	30 (100.00)	31 (100.00)	> 0.99^b^
Sneezing *n* (%)	0 (0.00)	1 (3.23)	> 0.99^b^
Clinical symptoms of otitis media			
Fever *n* (%)	12 (40.00)	14 (45.16)	0.68^a^
Purulent discharge from the ear canal (otorrhea) *n* (%)	28 (93.33)	31 (100.00)	0.24^b^
Earache *n* (%)	26 (86.67)	30 (96.77)	0.20^b^
Poor response to sound *n* (%)	9 (30.00)	10 (32.26)	0.85^a^
Vomiting or diarrhea *n* (%)	11 (36.67)	8 (25.81)	0.36^a^
Loss of appetite *n* (%)	17 (56.67)	17 (54.84)	0.89^a^
Nasal endoscopy			
Mucosal edema *n* (%)	30 (100.00)	31 (100.00)	> 0.99^b^
Mucopurulent discharge from middle meatus *n* (%)	30 (100.00)	31 (100.00)	> 0.99^b^
Ear endoscopy			
Tympanic membrane hyperemia *n* (%)	29 (96.67)	30 (96.77)	> 0.99^b^
Swollen, cloudy, loss of the bright triangle in the tympanic membrane *n* (%)	17 (56.67)	14 (45.16)	0.37^a^
Purulent discharge with a perforated tympanic membrane *n* (%)	30 (100.00)	31 (100.00)	> 0.99^b^
Perforated tympanic membrane with no purulent discharge *n* (%)	0 (0.00)	0 (0.00)	-
Positive for disease-causing bacteria			
*S. pneumoniae*	10 (33.33)	10 (32.26)	0.93^a^
*H. influenzae*	11 (36.67)	14 (45.16)	0.50^a^
*M. catarrhalis*	2 (6.67)	2 (6.67)	> 0.99^b^
Co-infection with 2 or 3 bacteria	5 (16.67)	5 (16.13)	0.95^a^

^a^Chi-S quare, ^b^Fisher’s Exact test.

### Symptom-relieving effects of nasal-spraying *Bacillus* spores

During the study period, no adverse events related to the nasal spray were reported among the participants. Specifically, there were no cases of nasal mucosal irritation, discomfort during administration, or systemic side effects such as nausea or vomiting. The product was well-tolerated by all the children, demonstrating its safety in pediatric patients with ARS and AOM.

**Fig. 2:**
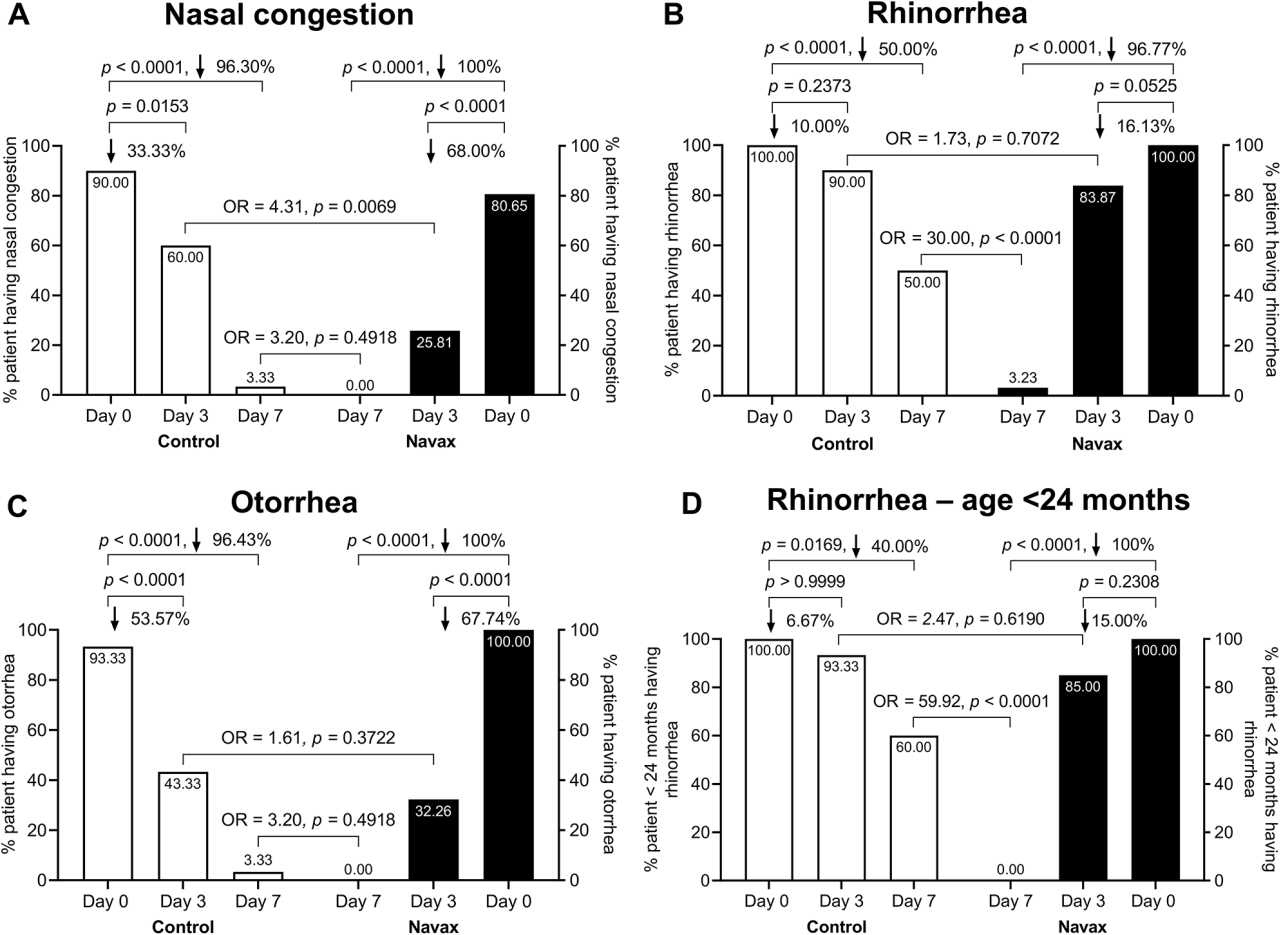
Percentages of patients exhibiting typical symptoms of acute rhinosinusitis and acute otitis media in the Control and Navax groups at days 0, 3, and 7: nasal congestion (**A**), rhinorrhea (**B**), otorrhea (**C**), and rhinorrhea in children < 24 months (**D**). The difference between data distribution was confirmed using the Chi-square and Fisher’s exact test.

To evaluate the effect of nasal-spraying *Bacillus* spore suspension as an adjunct to standard care in the treatment of rhinosinusitis and otitis media, we examined the percentage of patients exhibiting typical clinical symptoms at days 3 and 7, compared to day 0 in both the Control and Navax groups (Fig. 2). The two most common symptoms—nasal congestion and rhinorrhea—were included in the analysis presented in Fig. 2A,B. While the rates of patients experiencing nasal congestion was significantly reduced at days 3 and 7 compared to day 0 in both groups; a 2.04-fold difference between the Navax group (68%) and Control (33.33%) reached statistical significance by day 3 (Fig. 2A), with an odds ratio (OR) of 4.31 (95% CI: 1.38–11.63; *p* = 0.0069). Regarding rhinorrhea symptoms (Fig. 2B), the Navax group showed a trend toward a 16% reduction in the rates of patients having this symptom at day 3 compared to day 0, approaching statistical significance (*p* = 0.0525), while the Control group showed a smaller, non-significant 10% reduction (*p* = 0.273). At day 7, the percentage of patients with rhinorrhea reached a significant level of difference between the two groups. Notably, the Navax group demonstrated a 1.94-fold higher rate of rhinorrhea resolution (96.77%) at day 7 compared to the Control group (50%), with an OR of 30.00 (95% CI: 4.46–325.40) and a significant difference (*p* < 0.0001). In terms of the otitis media-associated otorrhea, while both groups presented a significant decrease during the two follow-up time points, the Navax group exhibiting a greater reduction than the Control group (*p* < 0.0001; Fig. 2C). Furthermore, the difference in rhinorrhea between the Navax and Control groups was shown to be more prominent in the infants (< 24 months) than in the older children subgroup (Fig. 2D and Fig. S1).

Other symptoms that revealed no significant inter-group difference included earache (Fig. S2), sneezing, fever, poor response to sound, vomiting, diarrhea, and loss of appetite (Table 1), which either resolved promptly, or expressed at too low rates before treatment.

**Fig. 3:**
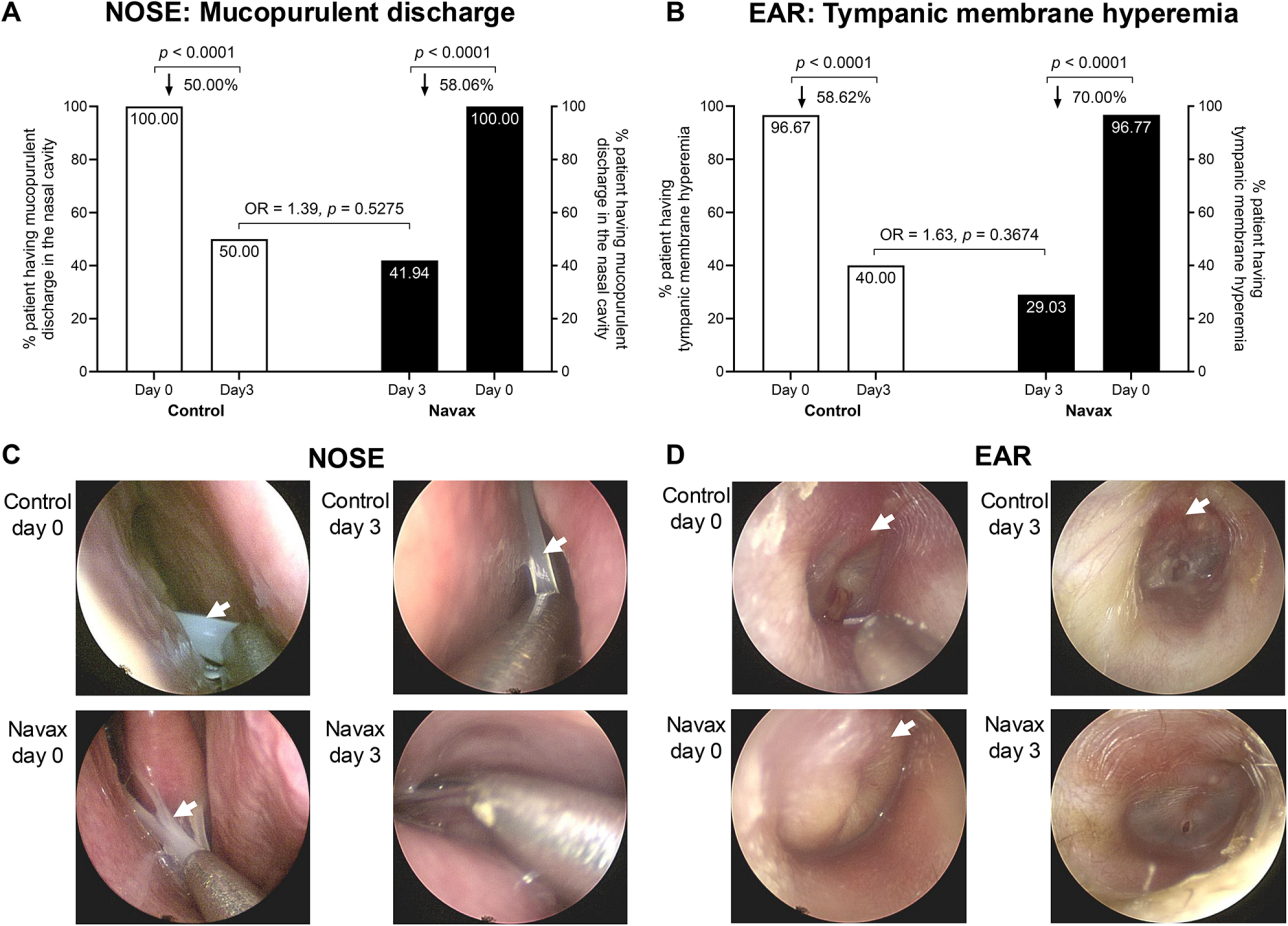
Endoscopic results demonstrating changes in nasal mucopurulent discharge and tympanic membrane hyperemia in the Control and Navax groups at day 3 compared to day 0. Percentages of patients exhibiting mucopurulent discharge from the middle meatus (**A**) and tympanic membrane hyperemia (**B**) in the Control and Navax groups at days 0 and 3. The difference between data distribution was confirmed using the Chi-square and Fisher’s exact test. Representative endoscopic images of the nose (**C**) and ear (**D**) show the locations of mucopurulent discharge from the middle meatus and tympanic membrane hyperemia (indicated by white arrows) in patients from the Control and Navax groups at days 0 and 3.

In addition to examining clinical symptoms, nasal and ear endoscopies were carried out to obtain direct images that help differentiate from other diseases with similar symptoms and support clinical signs (Fig. 3). The imaging analyses confirmed features of nasal mucopurulent discharge from the middle meatus and tympanic membrane hyperemia, which were rapidly reduced by day 3 in the Navax and Control groups (Fig. 3A,B; *p* < 0.0001). Although reductions in nasal mucopurulent discharge and tympanic membrane hyperemia were 8% (58.06% vs. 50.00%) and 11% (70.00% vs. 58.62%) greater in the Navax group compared to the Control group, with some patients in the Navax group showing complete resolution of discharge or hyperemia while those in the Control having no complete resolution (Fig. 3C,D), the overall quantitative analysis revealed no significant difference between the two groups.

### Lowering co-infecting bacteria load by nasal-spraying *Bacillus* spores

**Fig. 4:**
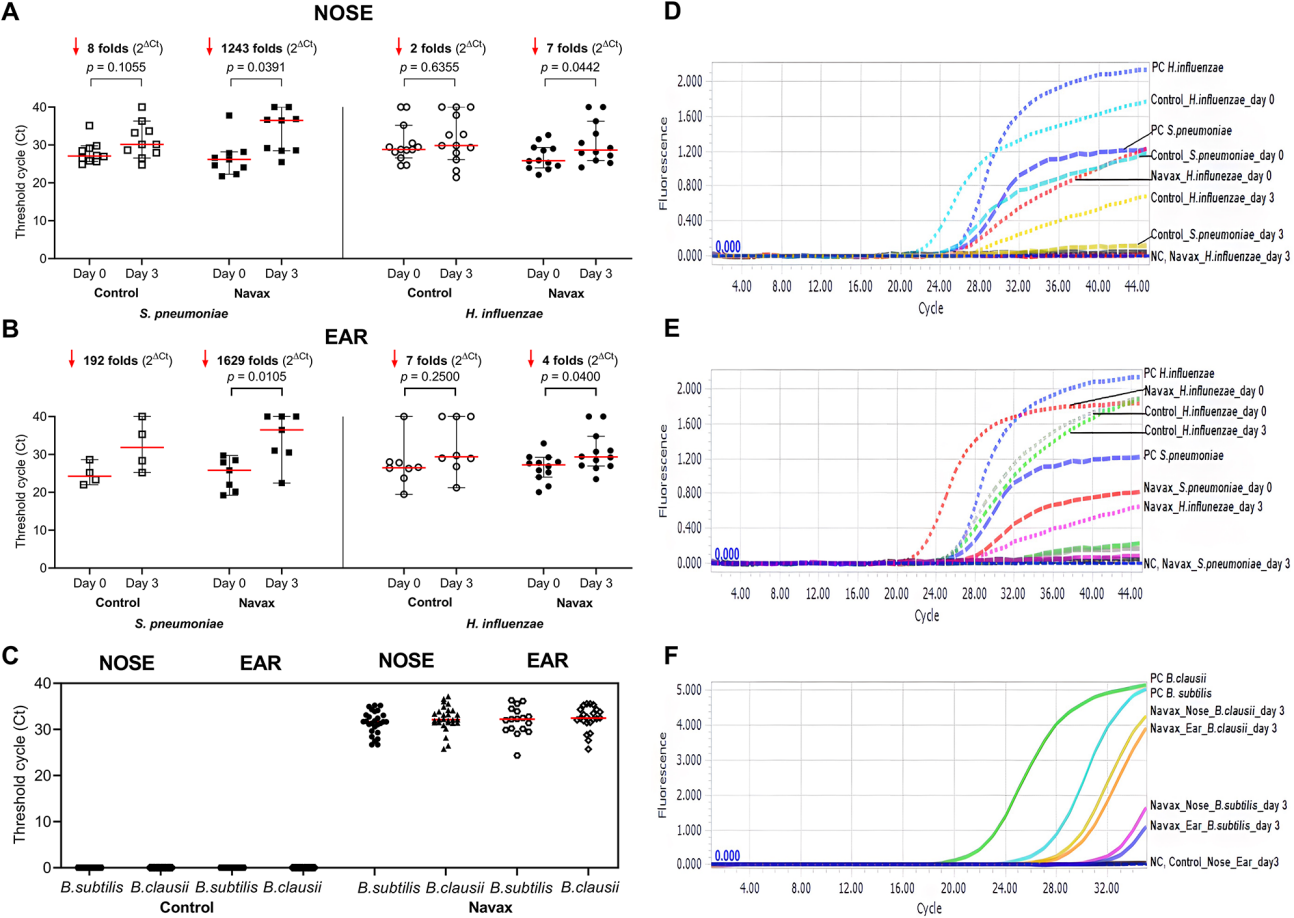
Real time PCR results showing C_t_ values, reducing-fold levels (2^ΔCt^), and amplification curves for *S. pneumoniae*, *H. influenzae*, *B. subtilis*, and *B. clausii* in nasopharyngeal (NOSE) and middle ear fluid (EAR) samples from Control and Navax groups at days 0 and 3. C_t_ and 2^ΔCt^ of fluorescence signals for *S. pneumoniae* and *H. influenzae* measured in samples of Control and Navax groups (**A**, **B**). C_t_ of fluorescence signals for *B. subtilis* and *B. clausii* measured in samples from the Control and Navax groups at day 3 (**C**). Real-time PCR TaqMan probe amplification curves for *S. pneumoniae* and *H. influenzae* from representative NOSE (**D**) and EAR (**E**) samples of the Control and Navax groups; real-time PCR SYBR Green amplification curves for *B. subtilis* and *B. clausii* (**F**) from samples of the Control and Navax groups at day 3; PC and NC are positive and negative controls. The Wilcoxon signed-rank test was used to determine the difference in C_t_ values of bacteria within a single group between days 0 and 3.

To explore the mechanisms by which *Bacillus* spores alleviate symptoms, we conducted real-time PCR with TaqMan probes to semi-quantitatively assess changes in the concentrations of co-infection bacteria, including the highly prevalent species *S. pneumoniae* and *H. influenzae*, in nasopharyngeal (NOSE) and middle ear fluid (EAR) samples. As a result, in the NOSE samples obtained from the Navax group, the C_t_ values, serving as reverse measurements of bacterial concentration, increased significantly at day 3 compared to day 0 for both *S. pneumoniae* (*p* = 0.0391) and *H. influenzae* (*p* = 0.0442), corresponding to a 1243-fold and 7-fold reductions, respectively, as calculated using the 2^ΔCt^ value. In contrast, such reduction of S. *pneumoniae* and *H. influenzae* in the Control group did not reach a significant level in NOSE samples (Fig. 4A), and similar results were obtained from EAR samples (Fig. 4B).

Figure 4D,E shows the amplification curves for *S. pneumoniae* and *H. influenzae* signals from representative NOSE and EAR samples in the Control and Navax groups. The data highlight significant reductions in co-infecting bacterial concentrations with Navax treatment, particularly as indicated by the absence of *H. influenzae* signals in the NOSE samples of the Navax group compared to the detectable signals in the Control group at day 3 (Fig. 4D), or the later appearance of *H. influenzae* signals in EAR samples of the Navax group compared to earlier signals in the Control group at day 3 (Fig. 4E).

To verify the proper use of 0.9% NaCl physiological saline in the Control group and LiveSpo Navax in the Navax group, and to address the question of whether *Bacillus* spores can migrate from the nose to the ear through nasal administration alone, we detected the presence of *B. subtilis* and *B. clausii* in both NOSE and EAR samples at days 0 and 3 via real-time PCR with SYBR Green. Notably, fluorescent signals indicating the presence of *B. subtilis* and *B. clausii* were clearly present in both sample types of the Navax group, but not in the Control group at day 3 (Fig. 4C,F). These results confirm that LiveSpo Navax was administered correctly and suggest that *B. subtilis* and *B. clausii* can migrate from the nasal cavity to the middle ear, effectively inhibiting the growth of co-infecting bacteria.

### Immunomodulatory activities of nasal-spraying *Bacillus* spores

To investigate the immunomodulatory effects of LiveSpo Navax, we evaluated changes in levels of common pro-inflammatory cytokines (IL-6, TNF-α, and IL-8) and immunoglobulin A (IgA) in the nasopharyngeal (NOSE) and middle ear fluid (EAR) samples after 3 days of treatment, when improvements in symptoms and a reduction in co-infecting bacteria were significantly observed.

**Fig. 5:**
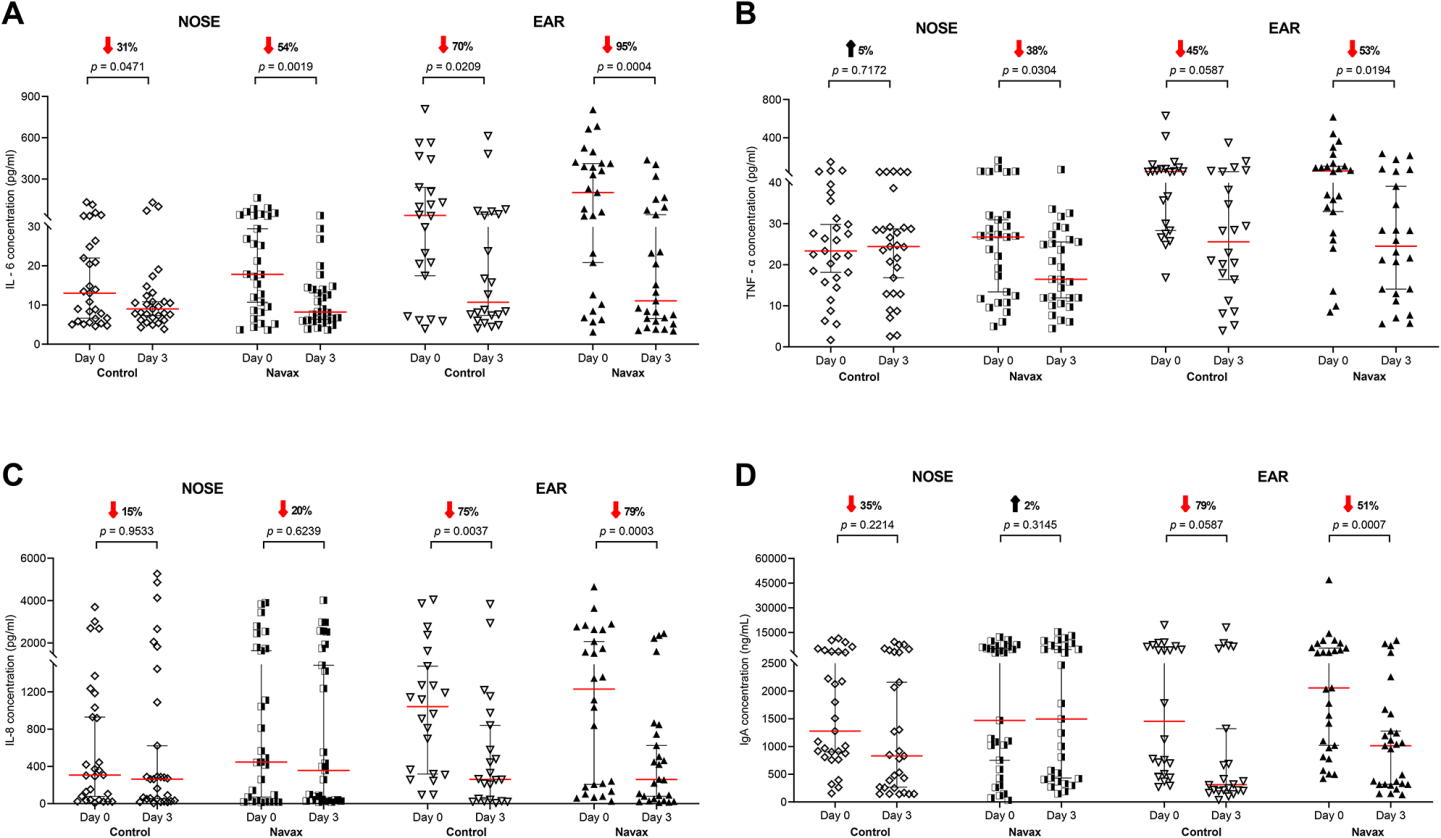
Pro-inflammatory cytokine levels (pg/mL) and immunoglobulin A (ng/mL) in nasopharyngeal (NOSE) and middle ear fluid (EAR) samples from the Control and Navax groups at day 3 compared to those at day 0. The Wilcoxon signed-rank test was used to calculate the median differences in IL-6 (**A**), TNF-α (**B**), IL-8 (**C**) and IgA (**D**) levels at days 0 and 3 in each group. The 95% CI for median in each group and the median difference between the two groups were shown in figure A–D. The significance level of all analyses was set at the *p* < 0.05.

As shown in Fig. 5A,B, the results observed in NOSE samples from the Navax group showed significant reductions in IL-6 and TNF-α levels by 54% (*p* = 0.0019) and 38% (*p* = 0.0304), respectively (day 0 vs. day 3: IL-6: 17.83 pg/mL vs. 8.20 pg/mL; TNF-α: 26.73 pg/mL vs. 16.46 pg/mL). In EAR samples, IL-6 and TNF-α levels were also reduced, by 95% (*p* = 0.0004) and 53% (*p* = 0.0194), respectively (day 0 vs. day 3: IL-6: 202.25 pg/mL vs. 11.07 pg/mL; TNF-α: 52.54 pg/mL vs. 24.50 pg/mL). In the Control group, IL-6 levels decreased in both NOSE and EAR samples but at a lower level, with reductions of 31% (*p* = 0.0471) in NOSE and 70% (*p* = 0.0209) in EAR samples. In contrast, TNF-α levels in NOSE samples from the Control group tended to increase, its reduction in EAR samples not statistically significant (*p* > 0.05). No significant changes in IL-8 levels were observed in either group or in either sample type (Fig. 5C).

The IgA levels tended to increase in NOSE samples of the Navax groups and decrease in the Control. In EAR samples, IgA decreased less in the Navax group than in the Control group (51% vs. 79%) (Fig. 5D). These data presented in Fig. 5 strongly suggest that nasal-spraying *Bacillus* spores effectively reduce pro-inflammatory cytokine levels and limit the decrease in IgA levels, thereby modulating the immune response against bacterial co-infection.

## Discussion

Although antibiotics are instrumental in management of pediatric AOM and ARS, particularly in cases with bacterial mucopurulent discharges, a serious dilemma is presented by emerging bacterial resistance to penicillin, ampicillin, trimethoprim/sulfamethoxazole, and macrolides in *S. pneumoniae* and *H. influenzae*^36–39^. In an approach to reduce antibiotic dependence and improve treatment for ARS and AOM, we used nasal-spraying *Bacillus* spore probiotics (LiveSpo Navax). The product proved safe and effective for supportive treatment of ARS and AOM, with no complications and significant reductions in symptoms, bacterial concentrations, and pro-inflammatory cytokines in nasopharyngeal and middle ear samples. Importantly, a faster (3–7 days) achievement of clinical effects (nasal congestion, rhinorrhea, and otorrhea) compared to extended treatment required for previously reported non-spore-forming probiotics^25–27,29–32,40–44^ was documented. Rapid effects of probiotics could contribute to a timely management of mucopurulent discharge and complications associated with spreading infection, an aim which still largely depends on antibiotics in the current practice.

The efficiency of a strategy using multi-strain spore-forming probiotics is further substantiated by their targeting species such as *S. pneumoniae* and *H. influenzae*, known as the key pathogens involved in ARS and AOM. Of note, LiveSpo Navax sprayed into the nose resulted in a reduction of pathogenic bacterial load in both the nasal cavity and middle ear, suggesting a Nasal-to-Ear transfer of the effect, a notion which is confirmed by detection of *B. subtilis* and *B. clausii* in the ear fluid samples post their nasal administration. It is likely that similar to pathogens’ ability to migrate via Eustachian tube to cause concurrent ARS and AOM, the probiotic *B. subtilis* and *B. clausii* may share the same route, but to fight pathogens.

Moreover, our findings also demonstrated that nasal-spraying *Bacillus* spores significantly reduced pro-inflammatory cytokine levels, specifically IL-6 and TNF-α, while preserving higher IgA levels than in the Control group. This modulation of the immune response may effectively counter the increase in bacterial co-infection in both the nasal and tympanic cavity, and aligns with otolaryngological endoscopy findings, which showed the reduction of nasal mucopurulent discharge and hyperemia of the TM. Elevated IL-6 and TNF-α are closely linked to rhinosinusitis severity and outcomes^45^. By reducing these cytokines, nasal-sparying *Bacillus* spores may attenuate inflammation, accelerate symptom resolution, and lower the risk of complications. Additionally, the observed reduction in these cytokines in the Navax group suggests a broader potential for *Bacillus* probiotics to modulate immune pathways involved in both acute and chronic conditions, as cytokine imbalances are also implicated in persistent inflammation and tissue remodelling^46^. IgA is the predominant immunoglobulin in the respiratory tract, playing a crucial role in defending the epithelial barrier from pathogens and regulating excessive immune responses during infections^47,48^. The maintenance of IgA levels in the nasal discharge of the Navax group could be attributed to the competitive action of *B. subtilis* and *B. clausii* against co-infecting bacteria on the mucosa, reducing pathogenic adhesion and invasion. We hypothesize that this outcome may be attributed to antibiotics reducing mucosal immune cells, including IgA-producing B cells^49^. This reduction could lead to decreased IgA production in the nasal mucosa of the Control group. In contrast, the antigenic surface proteins of *Bacillus* spores may stimulate mucosal IgA secretion, helping to prevent IgA decline in the nasal tract, enhancing defense against bacterial co-infection, and regulating excessive immune system activation. Our study yielded immunological data similar to those reported by Hishiki et al.^48^, showing that the use of probiotics helped maintain higher sIgA levels, particularly supporting the immune response in children with low intake of fermented foods.

The limitations of our study include delays due to the COVID-19 lockdown and social distancing policies in northern Vietnam, which postponed the start of the study from August 2021 to July 2022. Moreover, only patients with acute otitis media (AOM) and a perforated tympanic membrane were recruited. While symptom improvement was faster in these patients, making symptom follow-up feasible for this outpatient clinical trial, recruitment was slower due to the lower incidence of perforated AOM. Additionally, the wide age range of participants (1 month to 12 years) may have masked age-specific differences in clinical symptom progression and treatment responses, as significant improvements were primarily observed in rhinorrhea among children under 24 months. Furthermore, since the study participants were outpatients, potential bias may have arisen from variability in adherence to the prescribed nasal spray administration. Another limitation lies in the assessment of co-infecting bacterial concentrations and immunological indicators in nasopharyngeal and middle ear fluid samples, which was conducted only at days 0 and 3, but not at day 7, owing to ethical concerns in research involving children. Finally, the small sample size of 30–31 patients per group may be insufficient to assess significant differences in odds ratios for AOM outcomes, especially since the nasal spray’s effectiveness for AOM may be less robust than for ARS. To address this limitation, future research will involve a larger sample size to enhance statistical power and provide more reliable conclusions about the effectiveness of nasal spray for alleviating ARS and AOM across different developmental stages. This approach is particularly important for identifying age-specific responses and optimizing dosage. Furthermore, a study with a larger sample size should incorporate a multivariate model analysis to better explain variations in clinical symptoms and sub-clinical indicators, thereby providing a more accurate evaluation of Navax’s effects under these two conditions. Finally, long-term studies are necessary to evaluate the potential of nasal-spraying *Bacillus* spore probiotics for preventing recurrent episodes of ARS and AOM.

## Conclusion

This is the first randomized, single-blind, and controlled clinical trial to demonstrate the safety and efficacy of nasal-spraying *Bacillus* spores (LiveSpo Navax) as a supportive treatment in children diagnosed with both acute rhinosinusitis and acute otitis media with otorrhea. Navax treatment not only quickly reduces typical clinical symptoms, such as nasal congestion, rhinorrhea, otorrhea, nasal mucopurulent discharge, and tympanic membrane hyperemia, within only 3–7 days, but also significantly decreases the presence of co-infecting bacteria, including *S. pneumoniae* and *H. influenzae*, in both nasal and middle ear at day 3 during treatment. A nasal-to-ear transfer of *Bacillus* probiotics is confirmed by their detection in the ear post the nasal application. Additionally, it reduces excessive expression of pro-inflammatory cytokines such as IL-6 and TNF-α, while maintaining local IgA levels in the nasal cavity and middle ear. These findings indicate that nasal-spraying *Bacillus* spore probiotics provide a promising dual-benefit treatment for both ARS and AOM by reducing bacterial co-infection and modulating the immune response through a single administration route. The findings of this study may serve as a valuable reference, highlighting the benefits of probiotics as a natural and safe alternative to antibiotics. Additionally, these results could encourage policymakers to support the inclusion of probiotics in clinical guidelines as part of strategies to address the growing challenge of antibiotic resistance.

## Methods

### Materials

Detailed information about LiveSpo Navax was described previously^34,35^. In brief, LiveSpo Navax, a nasal-spray probiotic from LiveSpo Pharma (Hanoi, Vietnam), contains *B. subtilis* ANA4 and *B. clausii* ANA39 spores in a 0.9% NaCl physiological saline suspension at ≥ 5 × 10⁹ CFU/5 mL. It is manufactured as a Class-A medical device under registration number 210,001,337/PCBA-HN and is approved by Hanoi Health Department, Ministry of Health, Vietnam. The appearances of LiveSpo Navax and the placebo saline solution were indistinguishable because of opaque plastic spray bottles. The control product was prepared from 0.9% NaCl intravenous infusion solution (B. Braun, Germany). Products were coded, and blinding was maintained for the children, their parents, and most doctors and wet-lab technicians. Only a small number of key personnel, including the Principal Investigator (PI) and the project secretary, were unblinded to ensure proper administration of the research project.

### Ethical issues

This study received ethics approval from the Ethics Committee in Medical Research of Thai Binh University of Medicine and Pharmacy (Decision No. 872/HĐĐĐ, dated 09 August 2021). It adhered to the ethical principles of the Helsinki statement, ICH GCP guidelines, and Vietnam Ministry of Health regulations. Furthermore, the study was conducted in accordance with the CONSORT 2010 statement to ensure transparent and comprehensive reporting^50^. The parents of the pediatric participants were provided with information about the study and agreed to sign the informed consent form for their children’s participation. The participants could withdraw at any time. The study was registered with ClinicalTrials.gov under the identifier NCT05804123 on April 7, 2023.

### Study design, sample size, and criteria for patient collection

This study employed a randomized, single-blind, controlled clinical trial design and extended over 21-month period, from July 2022 to March 2024, at Department of Otolaryngology, Thai Binh Medical University Hospital, and Thai Binh Pediatric Hospital. The study’s patient cohort size (*n* = 30 per group) was determined based on the hypothesis that LiveSpo Navax treatment alleviates symptoms of ARS and AOM by 30% more effectively than the Control treatment (standard care), with a significance level of alpha = 0.05 and a power level of 0.8^34,35^. In fact, 41 patients per group, aged 1 month to 12 years, were enrolled to account for the high potential dropout rate (25%) during outpatient treatment, which was anticipated to be the COVID-19 pandemic occurring during the design of the clinical trial. As shown in Fig. 1, a total of 190 patients were screened for eligibility based on inclusion and exclusion criterias. The inclusion criteria included: (1) patients under 12 years of age, diagnosed with acute rhinosinusitis, such as nasal blockage/obstruction/congestion or discolored nasal discharge, or cough (daytime and night-time) for < 12 weeks; (2) patients who were also diagnosed with acute otitis media such as suffer from onset ear discharge not caused by otitis external, the TM was ruptured with purulent; (3) patients who were hospitalized or treated as outpatients but need periodic re-examination; (4) patients who had complete medical records or medical examination books; and (5) patients under 18 years old and whose parents agreed to participate in the study, explained, and signed the consent form. On the other hand, the exclusion criteria were as follows: (1) patients who did not agree to participate in the study; (2) did not have enough medical records or medical examination books; (3) patients moved out of the treatment unit (not for professional reasons); (4) outpatients had no periodic re-examination; (5) patients with congenital deafness, or deafness due to neurological causes (e.g. meningitis, obstetric complications, ear poisoning); and (6) patients with congenital disease-causing disorders of maxillofacial development and mental and physical retardation. The parents or guardians of patients were asked to provide the following information: full name, sex, age, address, and other details for demographic purposes.

### Randomization and interventions

A total of 82 participants were randomly assigned (1:1) to the Navax group (received LiveSpo Navax) or the Control group (received 0.9% NaCl). Participant allocation was randomized through a lottery process. Coded paper sheets marked with either number 1 or 2 were randomly selected from a container by a key investigator. These coded numbers were assigned to participants immediately after the informed consent form was signed by the parents of the patients. The coding numbers 1 and 2 corresponded to the Control and Navax groups. The flow charge of the study was shown in Fig. 1. The duration of standard treatment typically lasts for at least 7 days but may be extended to 14 days or longer, depending on the severity of the illness and the patient’s response to the treatment protocol. Nasal sprays were used in conjunction with standard hospital treatments, including single or combined oral, injectable, or ear drop antibiotics, and expectorants. In addition to the medications, procedures such as nasal irrigation and ear cleaning are also part of the treatment regimen, applied before nasal spraying. Parents or guardians of pediatric patients were given coded nasal sprays as blind samples to ensure objectivity and were trained on proper use. They were instructed to apply three sprays (50 µl each) into each nostril, three times daily, for at least 7 consecutive days, with possible extension during antibiotic treatment.

### Outcomes and assessments

#### Primary outcomes

Focused on changes in the percentages of patients having typical ARS and AOM symptoms at days 3 and 7 (during treatment) compared to day 0 (before treatment). ARS symptoms included nasal congestion, runny nose (rhinorrhea), and sneezing. AOM symptoms included fever, mucopurulent discharge from the ear canal (otorrhea), earache (indicated by rubbing or pulling on the earlobe, restlessness or tossing and turning during sleep, difficulty sleeping or crying), headache, temporary hearing loss (poor response to sound: a child’s weak or absent reaction to auditory stimuli, such as not turning their head when called or hearing clapping), as well as episodes of vomiting or diarrhea. Clinical symptoms were routinely assessed by doctors through interviews with the children’s parents. For older children, direct interviews were also conducted to enhance reliability. These symptoms were recorded in the medical records by doctors at days 0, 3, and 7, depending on the outpatients’ adherence to follow-up visits. In addition to clinical assessment, nasal and ear endoscopy was applied for observation of mucopurulent discharge and hyperemia of the tympanic membrane at days 0 and 3. Endoscopic evaluation of the ear, nose, and throat was performed using a 3.0 mm, 0-degree endoscope to examine the ear canal, tympanic membrane, ear discharge, nasal mucosa, nasal turbinates, middle meatus, and nasal secretions. There were no differences in symptom evaluation via endoscopy between infants and older children, except for the examination position: infants were held upright by their parents, whereas older children sat independently during the procedure. The examination of symptoms and endoscopy was performed according to the standard procedures of Thai Binh Medical University Hospital and Thai Binh Pediatric Hospital. Individual medical records were collected and systematically organized for data analysis.

#### Secondary outcomes

Included changes in nasopharyngeal and middle ear fluid samples for the following sub-clinical indicators: (1) concentrations of eight major co-infecting bacteria in the respiratory tract, including *Bordetella parapertussis*, *Bordetella pertussis*, *Chlamydophila pneumoniae*, *Haemophilus influenzae*, *Legionella pneumophila*, *Mycoplasma pneumoniae*, and *Streptococcus pneumoniae*, and *Moraxella catarrhalis*; (2) levels of pro-inflammatory cytokine, including IL-6, IL-8, and TNF-α; and (3) IgA levels. These assessments were conducted via following assays:

#### Real‑time PCR for detection of co-infecting bacteria

Nasopharyngeal fluid samples were collected from each participant at days 0, 3, and/or 7, while middle ear fluid samples were collected only at days 0 and 3. These samples were suspended in 0.9% NaCl physiological saline and used for DNA extraction. A total of 200 µl of specimen, processed in two replicates, was extracted using QIAamp DNA Mini Kit (Qiagen, MD, US), following the manufacturer’s guidelines. The resulting purified DNA (100 µl) was aliquoted into three PCR tubes (approximately 30 µl per tube) and stored at -80 °C. Purified DNA was used as the template for two real-time PCR assays. The first assay employed the Allplex Respiratory Panel 4 kit (SeeGene, Seoul, Korea) to concurrently identify seven bacteria associated with respiratory tract infections: *B. parapertussis*, *B. pertussis*, *C. pneumoniae*, *H. influenzae*, *L. pneumophila*, *M. pneumoniae*, and *S. pneumoniae*. The second assay used an in-house master mix for *M. catarrhalis* with primers and a probe as previously described^51^. The reaction conditions for both real-time PCR assays followed the company’s protocol: an initial denaturation at 95 °C for 15 min, followed by 45 cycles amplification and detection cycles at 95 °C for 10 s, 60 °C for 1 min, and 72 °C for 10 s. The analysis read-out was standardized to consider a C_t_ (threshold cycle) value of ≤ 30 as a positive result for the patient, since a C_t_ value > 30 indicates a low bacterial concentration that is insufficient to cause symptoms or is of no clinical significance. The real-time PCR protocol was conducted in accordance with the ISO 15189:2012 standard at the National Children’s Hospital. The reduction in bacterial concentrations was calculated using 2^ΔCt^, where the ΔC_t_ for target genes is determined by subtracting the C_t_ at day 3 from the C_t_ at day 0.

#### ELISA assays for determination of cytokine and IgA levels

Pro-inflammatory cytokine (IL-6, IL-8, and TNF-α) and IgA levels in nasopharyngeal and middle ear fluid samples were measured at days 0 and 3 using enzyme-linked immunosorbent assay (ELISA) kits according to the manufacturer’s guidelines and as described previously^34,35^. In brief, IL-6, IL-8, and TNF-α were quantified in pg/mL from 100 µL samples using the Human IL-6 DuoSet ELISA, Human IL-8 DuoSet ELISA, and Human TNF-α ELISA kits (R&D Systems, MN, US), respectively. IgA levels, measured in ng/mL, were determined using the Human IgA Uncoated ELISA kit (Thermo Fisher Scientific, MA, US).

#### Real‑time PCR for detection of *B. subtilis* and *B. clausii*

The detection of *B. subtilis* ANA4 and *B. clausii* ANA39 in nasopharyngeal and middle ear fluid samples was conducted for croos-check purposes at days 0, 3, and/or 7 using real-time PCR SYBR Green, following previous study^34,35^. In brief, the PCR conditions included an initial denaturation at 95 °C for 10 min, followed by 40 cycles of amplification at 95 °C for 15 s, 60 °C for 20 s, and 72 °C for 30 s. The results were analyzed based on a read-out standardization, with a C_t_ value of < 35 considered a confirmation of actual positive presence. The standardized protocol was routinely conducted at the Spobiotic Research Center, ANABIO R&D Ltd.

### Data collection and statistical analysis

Participant confidentiality and privacy were strictly maintained by the investigators, their staff, and the sponsor. Personal data, including clinical and sub-clinical information, were securely handled and accessible only to the PI and the project secretary directly involved in the study. At the end of the study, all records and research data for statistical analysis were securely stored at the administration office of Thai Binh University of Medicine and Pharmacy. Data were de-identified using unique study identification numbers to ensure that no identifying participant information was included. The data management systems were password-protected, and all databases were securely archived after the study. No data were shared with unauthorized third parties.

Tabular analysis was conducted for dichotomous variables using the χ^2^ or Fisher’s exact test when any cell’s expected value is below five. Continuous variables were compared using the Wilcoxon signed-rank or Mann-Whitney test in case of non-normally distributed data, and the t test for normally distributed data. Statistical and graphical analyses were performed using GraphPad Prism v8.4.3 software (GraphPad Software, CA, US). The significance level for all analyses was set at *p* < 0.05, *p*-values are used to determine statistical significance.

## Electronic supplementary material

Below is the link to the electronic supplementary material.

Supplementary Material 1

## Data Availability

The study protocol and datasets analysed during the current study are available from the corresponding authors on reasonable request.

## References

[1] Sakulchit, T. & Goldman, R. D. Antibiotic therapy for children with acute otitis media. *Can. Fam. Physician.* **63**, 685–687 (2017). 28904032 PMC5597011

[2] Zernotti, M. E. et al. Otitis media with effusion and atopy: Is there a causal relationship?. *World Allergy Organ J.* **10**, 37. 10.1186/s40413-017-0168-x (2017). 29158869 10.1186/s40413-017-0168-x PMC5684754

[3] Rettig, E. M. & Tunkel, D. E. In *Infections of the Ears, Nose, Throat, and Sinuses* (eds Durand, M. L. & Deschler, D. G.) 45–55 (Springer, 2018).

[4] Global Burden of Disease Study C. Global, regional, and national incidence, prevalence, and years lived with disability for 301 acute and chronic diseases and injuries in 188 countries, 1990–2013: A systematic analysis for the Global Burden of Disease Study 2013. *Lancet* **386**, 743–800. 10.1016/S0140-6736(15)60692-4 (2015). 26063472 10.1016/S0140-6736(15)60692-4 PMC4561509

[5] Nokso-Koivisto, J., Marom, T. & Chonmaitree, T. Importance of viruses in acute otitis media. *Curr. Opin. Pediatr.* **27**, 110–115. 10.1097/MOP.0000000000000184 (2015). 25514574 10.1097/MOP.0000000000000184 PMC4383320

[6] Hayashi, T. et al. Clinical practice guidelines for the diagnosis and management of acute otitis media in children-2018 update. *Auris Nasus. Larynx* **47**, 493–526. 10.1016/j.anl.2020.05.019 (2020). 32576390 10.1016/j.anl.2020.05.019

[7] Ngo, C. C., Massa, H. M., Thornton, R. B. & Cripps, A. W. Predominant bacteria detected from the middle ear fluid of children experiencing otitis media: A systematic review. *PLoS One* **11**, e0150949. 10.1371/journal.pone.0150949 (2016). 26953891 10.1371/journal.pone.0150949 PMC4783106

[8] Hu, Y. L. et al. Predominant role of Haemophilus influenzae in the association of conjunctivitis, acute otitis media and acute bacterial paranasal sinusitis in children. *Sci. Rep.* **11**, 11. 10.1038/s41598-020-79680-6 (2021). 33420151 10.1038/s41598-020-79680-6 PMC7794412

[9] DeMuri, G. P. & Wald, E. R. Clinical practice. Acute bacterial sinusitis in children. *N. Engl. J. Med.* **367**, 1128–1134. 10.1056/NEJMcp1106638 (2012). 22992076 10.1056/NEJMcp1106638

[10] Fokkens, W. J. et al. European position paper on rhinosinusitis and nasal polyps 2020. *Rhinology* **58**, 1–464. 10.4193/Rhin20.600 (2020). 32077450 10.4193/Rhin20.600

[11] Pagella, F., Colombo, A., Gatti, O., Giourgos, G. & Matti, E. Rhinosinusitis and otitis media: The link with adenoids. *Int. J. Immunopathol. Pharmacol.* **23**, 38–40 (2010). 20152078

[12] Wald, E. R. Acute otitis media and acute bacterial sinusitis. *Clin. Infect. Dis.* **52**(Suppl 4), S277-283. 10.1093/cid/cir042 (2011). 21460285 10.1093/cid/cir042 PMC7107845

[13] Venekamp, R. P., Sanders, S. L., Glasziou, P. P., Del Mar, C. B. & Rovers, M. M. Antibiotics for acute otitis media in children. *Cochrane Database Syst. Rev.* **2015**, CD000219. 10.1002/14651858.CD000219.pub4 (2015). 23440776 10.1002/14651858.CD000219.pub3

[14] Jaume, F., Valls-Mateus, M. & Mullol, J. Common cold and acute rhinosinusitis: Up-to-date management in 2020. *Curr. Allergy Asthma Rep.* **20**, 28. 10.1007/s11882-020-00917-5 (2020). 32495003 10.1007/s11882-020-00917-5 PMC7266914

[15] Suzuki, H. G., Dewez, J. E., Nijman, R. G. & Yeung, S. Clinical practice guidelines for acute otitis media in children: A systematic review and appraisal of European national guidelines. *BMJ Open* **10**, e035343. 10.1136/bmjopen-2019-035343 (2020). 32371515 10.1136/bmjopen-2019-035343 PMC7228535

[16] Ell, S. R. & Gan, R. W. C. In *Contemporary Rhinology: Science and Practice* (eds Swift, A. C., Carrie, S. & de Souza, C.) 287–299 (Springer, 2023).

[17] Frost, H. M., Becker, L. F., Knepper, B. C., Shihadeh, K. C. & Jenkins, T. C. Antibiotic prescribing patterns for acute otitis media for children 2 years and older. *J. Pediatr.* **220**, 109–115. 10.1016/j.jpeds.2020.01.045 (2020). 32111379 10.1016/j.jpeds.2020.01.045 PMC7249267

[18] Lemiengre, M. B. et al. Antibiotics for acute rhinosinusitis in adults. *Cochrane Database Syst. Rev.* **9**, CD006089. 10.1002/14651858.CD006089.pub5 (2018). 30198548 10.1002/14651858.CD006089.pub5 PMC6513448

[19] Imre, A., Ozturkcan, S. & Kalogjera, L. in *All Around the Nose: Basic Science, Diseases and Surgical Management* (eds Cingi, C. & Bayar Muluk, N.) 203–211 (Springer, 2020).

[20] Uddin, T. M. et al. Antibiotic resistance in microbes: History, mechanisms, therapeutic strategies and future prospects. *J. Infect Public Health* **14**, 1750–1766. 10.1016/j.jiph.2021.10.020 (2021). 34756812 10.1016/j.jiph.2021.10.020

[21] Goossens, H., Ferech, M., Vander Stichele, R. & Elseviers, M. Outpatient antibiotic use in Europe and association with resistance: A cross-national database study. *Lancet* **365**, 579–587. 10.1016/s0140-6736(05)17907-0 (2005). 15708101 10.1016/S0140-6736(05)17907-0

[22] Olivia, L. D. *WHO publishes list of bacteria for which new antibiotics are urgently needed*, <https://www.who.int/en/news-room/detail/27-02-2017-who-publishes-list-of-bacteria-for-which-new-antibiotics-are-urgently-needed> (2017).

[23] Laursen, R. P. & Hojsak, I. Probiotics for respiratory tract infections in children attending day care centers-a systematic review. *Eur. J. Pediatr.* **177**, 979–994. 10.1007/s00431-018-3167-1 (2018). 29752587 10.1007/s00431-018-3167-1

[24] Elshaghabee, F. M. F. & Rokana, N. Mitigation of antibiotic resistance using probiotics, prebiotics and synbiotics. A review. *Environ. Chem. Lett.* **20**, 1295–1308. 10.1007/s10311-021-01382-w (2022).

[25] Mukerji, S. S. et al. Probiotics as adjunctive treatment for chronic rhinosinusitis: A randomized controlled trial. *Otolaryngol. Head Neck Surg.* **140**, 202–208. 10.1016/j.otohns.2008.11.020 (2009). 19201289 10.1016/j.otohns.2008.11.020

[26] Habermann, W., Zimmermann, K., Skarabis, H., Kunze, R. & Rusch, V. Reduction of acute recurrence in patients with chronic recurrent hypertrophic sinusitis by treatment with a bacterial immunostimulant (*Enterococcus faecalis* Bacteriae of human origin. *Arzneimittelforschung* **52**, 622–627. 10.1055/s-0031-1299941 (2002). 12236051 10.1055/s-0031-1299941

[27] Endam, L. M. et al. Intranasal application of *Lactococcus lactis* W136 is safe in chronic rhinosinusitis patients with previous sinus surgery. *Front. Cell Infect. Microbiol.* **10**, 440. 10.3389/fcimb.2020.00440 (2020). 33154953 10.3389/fcimb.2020.00440 PMC7586919

[28] Scott, A. M. et al. Probiotics for preventing acute otitis media in children. *Cochrane Database Syst. Rev.* **6**, CD012941. 10.1002/14651858.CD012941.pub2 (2019). 31210358 10.1002/14651858.CD012941.pub2 PMC6580359

[29] Cardenas, N. et al. Prevention of recurrent acute otitis media in children through the use of *Lactobacillus salivarius* PS7, a target-specific probiotic strain. *Nutrients* **11**, 376. 10.3390/nu11020376 (2019). 30759799 10.3390/nu11020376 PMC6413216

[30] La Mantia, I. et al. Probiotics in the add-on treatment of rhinosinusitis: A clinical experience. *J. Biol. Regul. Homeost. Agents* **34**, 27–34 (2020). 33426863

[31] Gelardi, M. et al. A probiotic mixture in patients with upper respiratory diseases: The point of view of the otorhinolaringologist. *J. Biol. Regul. Homeost. Agents.* **34**, 5–10 (2020). 33426860

[32] Gelardi, M. et al. Probiotics in the add-on treatment of otitis media in clinical practice. *J. Biol. Regul. Homeost. Agents.* **34**, 19–26 (2020). 33426862

[33] Golnari, M. et al. Isolation and characterization of novel *Bacillus* strains with superior probiotic potential: Comparative analysis and safety evaluation. *Sci. Rep.* **14**, 1457. 10.1038/s41598-024-51823-z (2024). 38228716 10.1038/s41598-024-51823-z PMC10791968

[34] Tran, D. M. et al. Nasal-spraying *Bacillus* spores as an effective symptomatic treatment for children with acute respiratory syncytial virus infection. *Sci. Rep.* **12**, 12402. 10.1038/s41598-022-16136-z (2022). 35858943 10.1038/s41598-022-16136-z PMC9297280

[35] Tran, T. T. et al. Efficient symptomatic treatment and viral load reduction for children with influenza virus infection by nasal-spraying *Bacillus* spore probiotics. *Sci. Rep.* **13**, 14789. 10.1038/s41598-023-41763-5 (2023). 37684332 10.1038/s41598-023-41763-5 PMC10491672

[36] Cillóniz, C., Garcia-Vidal, C., Ceccato, A. & Torres, A. In *Antimicrobial Resistance in the 21st Century* Ch. Chapter 2, 13–38 (Springer, 2018).

[37] Mohanty, S., Johnson, K. D., Yu, K. C., Watts, J. A. & Gupta, V. A multicenter evaluation of trends in antimicrobial resistance among streptococcus pneumoniae isolates from adults in the United States. *Open Forum Infect. Dis.* **9**, ofac420. 10.1093/ofid/ofac420 (2022). 36168549 10.1093/ofid/ofac420 PMC9511122

[38] Armbruster, C. E. et al. Indirect pathogenicity of *Haemophilus influenzae* and *Moraxella catarrhalis* in polymicrobial otitis media occurs via interspecies quorum signaling. *mBio*. **1**, 10.1128/mBio.00102-10 (2010). 10.1128/mBio.00102-10 PMC2925075 20802829

[39] Jacobs, M. R. in *Antimicrobial Drug Resistance* Ch. Chapter 7, 867–888 (Springer, 2017).

[40] Skovbjerg, S. et al. Spray bacteriotherapy decreases middle ear fluid in children with secretory otitis media. *Arch. Dis. Child.* **94**, 92–98. 10.1136/adc.2008.137414 (2009). 18713796 10.1136/adc.2008.137414

[41] Marchisio, P., Esposito, S. & Principi, N. The evidence for applying *Streptococcus salivarius* 24SMB by nasal spray for preventing recurrent acute otitis media. *Eur. J. Clin. Microbiol. Infect. Dis.* **35**, 1889–1890. 10.1007/s10096-016-2729-2 (2016). 27461220 10.1007/s10096-016-2729-2

[42] La Mantia, I., Varricchio, A. & Ciprandi, G. Bacteriotherapy with *Streptococcus salivarius* 24SMB and *Streptococcus oralis* 89a nasal spray for preventing recurrent acute otitis media in children: A real-life clinical experience. *Int. J. Gen. Med.* **10**, 171–175. 10.2147/IJGM.S137614 (2017). 28684920 10.2147/IJGM.S137614 PMC5484566

[43] Passali, D. et al. The efficacy and tolerability of *Streptococcus salivarius* 24SMB and *Streptococcus oralis* 89a administered as nasal spray in the treatment of recurrent upper respiratory tract infections in children. *Eur. Rev. Med. Pharmacol. Sci.* **23**, 67–72. 10.26355/eurrev_201903_17352 (2019). 30920629 10.26355/eurrev_201903_17352

[44] Manti, S. et al. Bacteriotherapy with *Streptococcus salivarius* 24SMB and *Streptococcus oralis* 89a nasal spray for treatment of upper respiratory tract infections in children: A pilot study on short-term efficacy. *Ital. J. Pediatr.* **46**, 42. 10.1186/s13052-020-0798-4 (2020). 32245500 10.1186/s13052-020-0798-4 PMC7126168

[45] Scheckenbach, K. & Wagenmann, M. Cytokine patterns and endotypes in acute and chronic rhinosinusitis. *Curr. Allergy Asthma Rep.* **16**, 3. 10.1007/s11882-015-0583-4 (2016). 26707380 10.1007/s11882-015-0583-4 PMC7088912

[46] Kato, A. et al. Evidence of a role for B cell-activating factor of the TNF family in the pathogenesis of chronic rhinosinusitis with nasal polyps. *J. Allergy Clin. Immunol.* **121**, 1385–1392. 10.1016/j.jaci.2008.03.002 (2008). 18410958 10.1016/j.jaci.2008.03.002 PMC2802261

[47] Olaimat, A. N. et al. The potential application of probiotics and prebiotics for the prevention and treatment of COVID-19. *NPJ Sci. Food* **4**, 17. 10.1038/s41538-020-00078-9 (2020). 33083549 10.1038/s41538-020-00078-9 PMC7536434

[48] Hishiki, H. et al. A double-blind, randomized, placebo-controlled trial of heat-killed *Pediococcus acidilactici* K15 for prevention of respiratory tract infections among preschool children. *Nutrients* **12**, 1989. 10.3390/nu12071989 (2020). 32635408 10.3390/nu12071989 PMC7400799

[49] Kuzin, I. I. et al. Tetracyclines inhibit activated B cell function. *Int. Immunol.* **13**, 921–931. 10.1093/intimm/13.7.921 (2001). 11431422 10.1093/intimm/13.7.921

[50] Moher, D. et al. CONSORT 2010 explanation and elaboration: Updated guidelines for reporting parallel group randomised trials. *BMJ* **340**, c869. 10.1136/bmj.c869 (2010). 20332511 10.1136/bmj.c869 PMC2844943

[51] Greiner, O., Day, P. J., Altwegg, M. & Nadal, D. Quantitative detection of *Moraxella catarrhalis* in nasopharyngeal secretions by real-time PCR. *J. Clin. Microbiol.* **41**, 1386–1390. 10.1128/JCM.41.4.1386-1390.2003 (2003). 12682118 10.1128/JCM.41.4.1386-1390.2003 PMC153888

