# Supplementary material for: Alleviating symptoms of paediatric acute rhinosinusitis and acute otitis media with otorrhea using nasal-spraying Bacillus probiotics: a randomized controlled trial
Source: Sci Rep. 2025 Jan 27;15:3410. doi: 10.1038/s41598-025-87372-2 (PMC11772584; doi:10.1038/s41598-025-87372-2)
Supplement: Supplementary file 1 — Supplementary Material 1 [file 41598_2025_87372_MOESM1_ESM.pdf]

Supplemental data

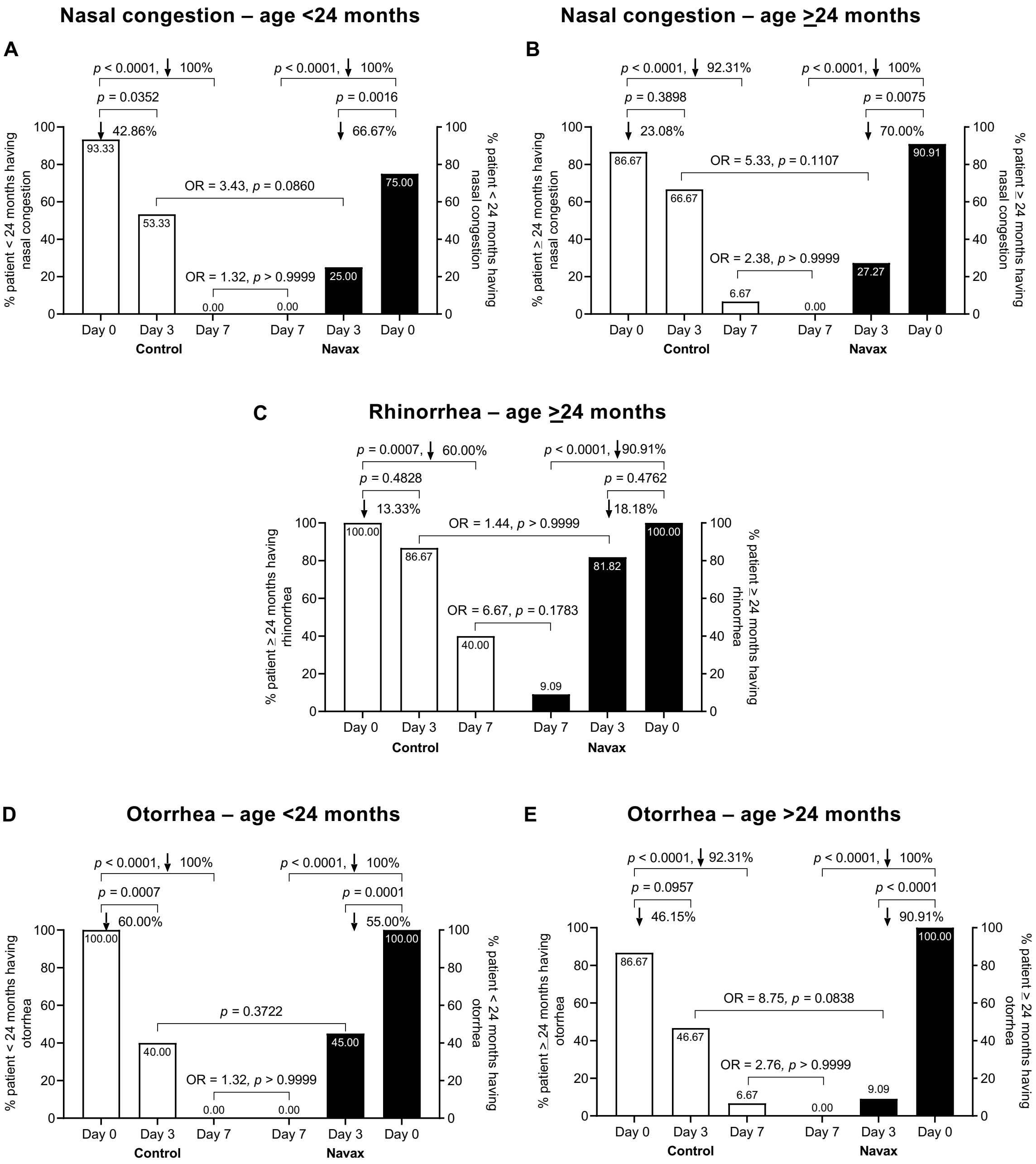

Fig. S1. Percentage of patients in the Control and Navax groups exhibiting typical symptoms of acute rhinosinusitis (A-B: nasal congestion and C: rhinorrhea) and acute otitis media (D-E: otorrhea), divided into two age subgroups (subgroup 1: 1 month to <24 months; subgroup 2: >2 to 12 years). Statistical significance was assessed using the Chi-square and Fisher's exact tests, highlighting differences in symptom reduction between groups over time.

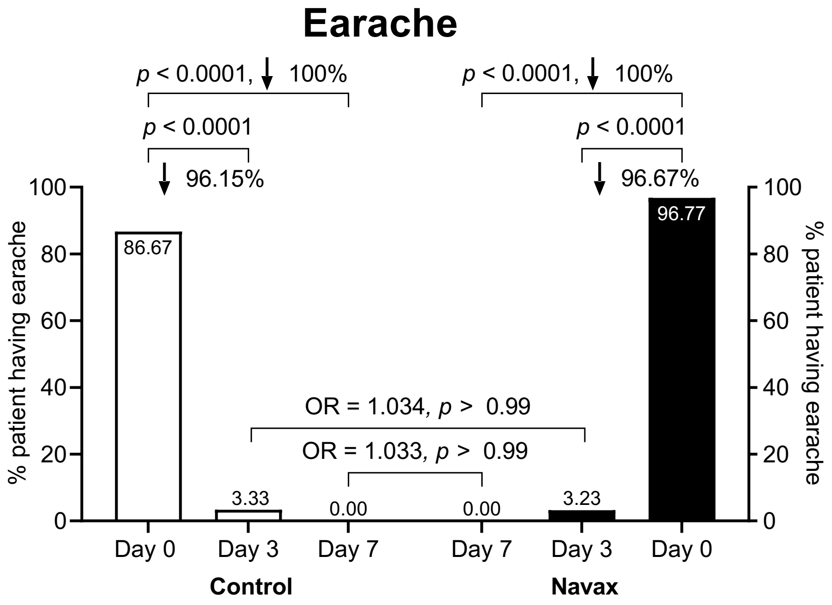

Fig. S2. Percentage of patients exhibiting earache symptom of acute otitis media in Control and Navax groups at days 0, 3, and 7. The difference between data distribution was confirmed using the Chi-square and Fisher's exact test.
